# Retinal Microvasculature Changes in Patients With Coronary Total Occlusion on Optical Coherence Tomography Angiography

**DOI:** 10.3389/fmed.2021.708491

**Published:** 2021-12-16

**Authors:** Pingting Zhong, Yijun Hu, Lei Jiang, Qingsheng Peng, Manqing Huang, Cong Li, Yu Kuang, Ning Tan, Honghua Yu, Xiaohong Yang

**Affiliations:** ^1^Department of Ophthalmology, Guangdong Eye Institute, Guangdong Provincial People's Hospital, Guangdong Academy of Medical Sciences, Guangzhou, China; ^2^State Key Laboratory of Ophthalmology, Guangdong Provincial Key Laboratory of Ophthalmology and Visual Science, Zhongshan Ophthalmic Center, Sun Yat-sen University, Guangzhou, China; ^3^Aier School of Ophthalmology, Central South University, Changsha, China; ^4^Aier Institute of Refractive Surgery, Refractive Surgery Center, Guangzhou Aier Eye Hospital, Guangzhou, China; ^5^Guangdong Geriatrics Institute, Guangdong Provincial People's Hospital, Guangdong Academy of Medical Sciences, Guangzhou, China; ^6^School of Medicine, South China University of Technology, Guangzhou, China; ^7^Guangdong Provincial Key Laboratory of Coronary Heart Disease Prevention, Department of Cardiology, Guangdong Cardiovascular Institute, Guangdong Provincial People's Hospital, Guangdong Academy of Medical Sciences, Guangzhou, China

**Keywords:** coronary artery disease, coronary total occlusion, optical coherence tomography angiography, retinal microvasculature, prediction

## Abstract

**Background:** Retinal microvasculature has been associated with coronary artery disease (CAD), but the exact contributory role in coronary total occlusion (CTO) is unclear. We aimed to investigate whether retinal vasculature is associated with CTO and could provide incremental value in the assessment of CTO.

**Methods:** A total of 218 CAD patients including 102 CTO and 116 non-CTO were enrolled. Retinal vasculature was measured by optical coherence tomography angiography (OCTA) for all patients. Receiver operating characteristic (ROC) curve was used to assess the performance of retinal vasculature in differentiating CTO from non-CTO patients.

**Results:** In non-CTO CAD patients, vessel density (VD) of mean superficial capillary plexus (SCP) and parafovea SCP were 49.85 and 52.56%, respectively; in CTO patients, VD of mean SCP and parafovea SCP were 47.77, and 49.58%, respectively. After multiple adjustment, VD in the SCP was significantly lower in CTO patients compared to non-CTO patients. VD of superior hemi in the parafovea SCP combined with the clinical variates showed the best ability to predict CTO from CAD with an area under the curve (AUC) of 0.812 (specificity of 89.0% and sensitivity of 65.9%).

**Conclusions:** In CTO patients, retinal VD was significantly decreased, and microvascular damage might specifically target to arterioles than capillaries. Retinal vasculature could thus be a surrogate for detecting the microvascular damage and assist in the assessment of CTO patients. OCTA examination could be suggested to monitor the process of coronary arteries lesions.

## Introduction

Coronary artery disease (CAD) is the leading cause of mortality in the world. It places an remarkable burden on the families and society (1). One of the most intractable situations of CAD is the development of chronic total occlusions (CTOs). CTO is a subgroup of coronary lesions, representing complete occlusions of coronary arteries with a thrombolysis in myocardial infarction (TIMI) flow of zero and an estimated duration of at least 3 months (2). The diagnosis of CTO is made based on coronary angiography (CAG). Besides, regardless of the continuous efforts and improvements of technology in interventional cardiology (3), the treatment outcome and prognosis of CTO remain unsatisfying with low success rate, high recurrence rate and mortality (4).

Many retinal indications have been demonstrated the usefulness in many specialties. For example, a previous study has illustrated the postoperative changes of retinal nerve fiber layer (RNFL) thickness appeared to be transient among the patients with open-angle glaucoma (5). Besides, the most profound toxic effects of ophthalmic complications have been presented in the cornea and retina in the patients with hyperglycemia or diabetes (6). Vasculature of the retina and the heart share many common properties in morphology and physiology (7). Because the retinal vessels can be observed directly, they may potentially represent a clinical method for non-invasively assessing systemic microcirculation (7). A number of studies have proved that the structural and functional changes of retinal vasculature, such as arteriolar narrowing, venular dilating, arteriovenous nipping, and vessel tortuosity, were associated with cardiovascular disorders (8–10). Furthermore, the value of retinal vasculature signs were also suggested to be predictive of a cardiovascular prognosis (10). Recently, retinal vessel density (VD) as measured by optical coherence tomography angiography (OCTA) has been explored for any associations with CAD (11, 12). OCTA is an ophthalmologic technique used to non-invasively visualize and accurately quantify the retinal vasculature. However, the quantitative analysis of retinal vasculature in CTO is poorly documented. Since CTO is not unusual at our clinic as a major cardiovascular institute in South China and many CAD patients are referred to here, we are quite interested in exploring the retinal vasculature changes in CTO patients.

In this study, OCTA was used to quantitatively measure retinal vasculature among CTO and non-CTO CAD patients. The present study was undertaken to explore the changes of retinal vasculature in CTO patients. We aimed to assess whether retinal vasculature could be a surrogate to reflect the microvasculature status and provide any value to assist in the assessment of CTO.

## Methods

### Design and Population of Study

This cross-sectional study was conducted in the Department of Cardiology of Guangdong Provincial People's Hospital (GDPH) from November 2018 to July 2019. All the CTO patients were hospitalized for percutaneous coronary intervention after being regularly assessed by primary cardiologists. The CTO patients were angiographically enrolled with ≥ 1 CTO in a main coronary artery. Besides, we also consecutively recruited non-CTO CAD patients angiographically confirmed with ≥ 50% stenosis in at least one major coronary artery. Overall, a total of 218 CAD patients including 102 CTO patients and 116 non-CTO patients were enrolled. All included patients successfully received an OCTA examination with interpretable data 1 day before CAG.

The exclusion criteria were (1) coexisting hemodynamic instability (hypertension or hypotension along with changes in heart rate); (2) pre-existing hypertensive or diabetic retinopathy (HR or DR, respectively) based on the medical history provided and the clinical examination results of slit-lamp biomicroscopy, indirect ophthalmoscopy, two 45-degree mydriatic color fundus photographs, and OCTA; (3) disability to cooperate with ophthalmic examinations; or (4) moderate or high refractive error (≥±3 diopters). We also excluded patients with prior revascularization. During the OCTA examination, a cardiologist was asked to monitor the patients' hemodynamic status.

The study was approved by the Research Ethics Committee of the hospital [No. GDREC2019554H(R1)] and was performed according to the Declaration of Helsinki. Patient consent was obtained.

### Data Extraction and Processing

Data were extracted from the hospital registration system and clinical records by trained research assistants. Cardiac data were documented in the forms of demographic, clinical and imaging. Prior to the OCTA and CAG procedure, a structural interview was performed to collect the baseline on the presence of cardiovascular risk factors in the patients, where in particular, significant cardiovascular risk factors such as age, sex, body mass index (BMI), history of hypertension and diabetes mellitus (DM), and baseline cholesterol values were documented. We also collected other cardiac data with high-sensitivity troponin T (hs-TnT), N terminal pro-brain natriuretic peptide (NT-proBNP) and left ventricular ejection fraction (LVEF). All laboratory analyses were done via routine blood tests within 3 days before the OCTA examination. The LVEF was measured by ultrasonography within the 24 h after admission.

### Retinal Images Assessment

OCTA (RTVue-XR Avanti; Optovue, Fremont, CA, USA) is a fully automated and fast (<1 min per eye) ophthalmic examination (13). The OCTA instrument is an 840-nm wavelength spectral-domain OCT, and it generates 304 × 304 scans in 2.9 s at a speed of 70 kHz (14, 15). The device uses a split-spectrum amplitude-decorrelation angiography (SSADA) algorithm (16).

The high-definition modes of optic disc (4.5 × 4.5 mm^2^) and macula (6 × 6 mm^2^) within the OCTA scans were performed for all included patients. The microvasculature parameters in the optic disc included the VD and RNFL thickness of the radial peripapillary capillary (RPC); the microvasculature parameters in the macula included the VD of the superficial capillary plexus (SCP), deep capillary plexus (DCP), and FD (foveal density) 300. In the disk area, RPC is a slab extending from the internal limiting membrane (ILM) to the RNFL that fits a circle (2 mm in diameter) centered on the optic disc. The peripapillary region is defined as a 1–2 mm round annulus around the optic disc, while the capillary density was measured with automatic remove of larger vessels (diameter ≥ 33 μm). In the macular region, SCP refers to a slab extending from the ILM to 10 μm above the inner plexiform layer (IPL), while the DCP slab extends from 10 μm above the IPL to 10 μm below the outer plexiform layer (OPL). Parafovea refers to the area between the 1–3 mm concentric ring center of the fovea. FD300 is a parameter demonstrating the capillary density from ILM to OPL in a 300-μm wide region around the foveal avascular zone (FAZ) (17).

Both eyes of the participants were examined by OCTA, but data from only one eye were used. For analysis, we used right eye data for participants born in even-numbered years and left eye data for those born in odd-numbered years. If the scan was uninterpretable for the selected eye, data from the other eye were included. Only images with a quality index ≥ 6/10 were retained.

### Coronary Arteries Lesions Assessment

All patients approached for this study were already internally and externally referred and consented to the invasive CAG procedure. Routine diagnostic CAG was performed via radial or femoral approach using a catheter of 6-Fr dimension. Two orthogonal views were examined in end-diastole to maximize contrast enhancement and vessel diameter for each major coronary artery. The image with the most severe stenosis was selected for each evaluated segment of the coronary arteries. All angiograms were analyzed by two trained cardiologists blinded to the results of adjunctive investigation and retinal assessment. In this study, the Gensini score was used to quantify the severity of CAD and the score calculation was done by two cardiologists using angiograms. The Gensini score has been previously described in detail (18).

### Statistical Analysis

SPSS software package version 20 (SPSS. Inc., Chicago, IL, USA) was used for statistical analysis. Means ± standard deviation (SD) were used for presenting quantitative variables with normal distribution, medians (interquartile range, IQR) for abnormal distribution. Numbers (percentages) were used for categorical variables. For comparisons of continuous data, an independent two-tailed Student's *t*-test or Mann-Whitney test was used, and for comparisons of categorical data, a χ^2^ test or Fischer's exact test was used.

In comparisons of OCTA parameters between non-CTO and CTO groups, baseline characteristics were adjusted. Confounding risk factors were assessed using Framingham risk factors included age, sex, total cholesterol (TC), high-density lipoprotein cholesterol (HDL-C), history of blood pressure (BP) grade, DM, and smoking. Besides, other potentially confounding risk factors were also adjusted: LVEF, hs-TnT, NT-proBNP, and Gensini score.

To figure out the performance of retinal vasculature in the discrimination of CTO from CAD, we conducted a several-steps analysis of the relationship between the presence of CTO and the risk factors. Receiver operating characteristic (ROC) analysis was carried out and area under the curve (AUC) was calculated. Odds ratio (OR), confidence interval (CI) stated at 95% and *p* value were expressed as outcomes. *p* < 0.05 was considered statistically significant.

## Results

Overall, the mean age of 57 years and males of 94 (81.0%) were for the non-CTO group, and the mean age of 58 years and males of 97 (95.1%) were for CTO group. Table 1 shows the clinical characteristics among the whole group.

**Table 1:** Demographic and clinical characteristics of included CAD patients (*n* = 218).

	**Overall**	**Non-CTO group**	**CTO group**	***p-*value**
	**(*n* = 218)**	**(*n* = 116)**	**(*n* = 102)**	
Age (years), mean ± SD	58.25 ± 8.6	57.74 ± 8.3	58.83 ± 8.9	0.352
Males, *n* (%)	191 (87.6)	94 (81.0)	97 (95.1)	0.002^*^
Hypertension, *n* (%)	116 (53.2)	61 (52.6)	55 (53.9)	0.844
BP grade, *n* (%)				0.793
Grade I (reference)	18 (8.3)	10 (8.6)	8 (7.8)	
Grade II	41 (18.8)	20 (17.2)	21 (20.6)	
Grade III	56 (25.7)	31 (26.7)	25 (24.5)	
Diabetic mellitus, *n* (%)	39 (17.9)	20 (17.2)	19 (18.6)	0.790
Smoker, *n* (%)	74 (33.9)	37 (31.9)	37 (36.3)	0.496
BMI (kg/m^2^), mean ± SD	24.51 ± 3.0	24.68 ± 3.1	24.31 ± 2.9	0.382
LVEF (%), median (IQR)	64.00 (58.00–68.00)	66.00 (63.00–69.00)	60.00 (45.00–65.25)	<0.001^*^
**Systemic treatments**, ***n*** **(%)**				
Beta- blockers	53 (24.3)	26 (22.4)	27 (26.5)	0.486
Calcium channel blocker	42 (19.3)	19 (16.4)	23 (22.5)	0.249
ACEI/ARBs	70 (32.1)	38 (32.8)	32 (31.4)	0.827
Aspirin	218 (100)	116 (100)	102 (100)	1.000
Clopidogrel	219 (100)	116 (100)	102 (100)	1.000
Statin	220 (100)	116 (100)	102 (100)	1.000
TC (mmol/L), median (IQR)	4.20 (3.55–5.17)	4.16 (3.66–5.16)	4.30 (3.48–5.17)	0.989
Triglycerides (mmol/L), median (IQR)	1.56 (1.17–2.14)	1.56 (1.17–2.16)	1.57 (1.17–2.14)	0.765
HDL-C (mmol/L), median (IQR)	0.95 (0.82–1.10)	0.93 (0.80–1.13)	0.95 (0.83–1.06)	0.732
LDL-C (mmol/L), median (IQR)	2.81 (2.21–3.55)	2.79 (2.22–3.51)	2.86 (2.14–3.56)	0.879
HbA_1_C (%), median (IQR)	5.90 (5.60–6.20)	5.80 (5.60–6.13)	5.90 (5.60–6.30)	0.317
hs-TnT (pg/ml), median (IQR)	12.45 (9.10–34.33)	10.25 (7.78–20.03)	18.15 (11.03–40.90)	<0.001^*^
NT-proBNP (pg/mL), median (IQR)	103.60 (46.15–395.55)	58.65 (30.88–120.28)	303.70 (83.30–674.20)	<0.001^*^
Gensini score, median (IQR)	54.00 (28.00–85.00)	33.50 (22.75–56.00)	97.00 (67.00–121.50)	<0.001^*^

*CAD, coronary artery disease; CTO, coronary total occlusion; BP, blood pressure; BMI, body mass index; LVEF, left ventricular ejection fraction; TC, total cholesterol; HDL-C, high-density lipoprotein cholesterol; LDL-C, low-density lipoprotein cholesterol; HbA_1_C, hemoglobin A_1_C; hs-TnT, high-sensitivity troponin T; NT-proBNP, N terminal pro-brain natriuretic peptide. The BP grade (SBP/DBP) were defined as: I = 140–159/90–99 mmHg; II = 160–179/100–109 mmHg; III = ≥ 180/110 mmHg*.

* *p < 0.05 is considered statistically significant*.

### The Difference of Retinal Vasculature Between the Two Groups

After multiple adjustment of age, sex, TC, HDL-C, history of BP grade, DM, and smoke, LVEF, hs-TnT, NT-proBNP and Gensini score, there was no significant difference in the RNFL thickness, VD of the RPC and capillary RPC between the comparisons of non-CTO and CTO groups.

In the macular VD, the significant difference only showed in the SCP between the comparison of non-CTO and CTO groups. In the non-CTO patients, VD of mean SCP and parafovea SCP were 49.85 and 52.56%, respectively; in CTO patients, VD of mean SCP and parafovea SCP were 47.77 and 49.58%, respectively. The VD in the SCP was significantly lower in in CTO group compared to non-CTO group (all *p* < 0.05) (see Table 2).

**Table 2:** The comparisons of retinal vasculature parameters in the study groups (*n* = 218).

**Retinal vasculature parameters**	**Overall**	**Non-CTO**	**CTO**	***p*-value^†^**
	**(*n* = 218)**	**(*n* = 116)**	**(*n* = 102)**	
**RNFL thickness (μm), mean** **±** **SD**				
Mean	114.52 ± 13.8	114.28 ± 11.5	114.79 ± 16.0	0.884
Superior	138.50 ± 23.3	137.86 ± 15.9	139.23 ± 29.7	0.799
Inferior	146.89 ± 18.7	147.05 ± 17.2	146.71 ± 20.4	0.748
Temporal	77.16 ± 10.6	77.02 ± 9.7	77.33 ± 11.6	0.881
Nasal	100.15 ± 17.8	99.95 ± 15.1	100.38 ± 20.5	0.418
**RPC density (%), mean** **±** **SD**				
Mean	55.68 ± 3.0	56.15 ± 2.6	55.14 ± 3.2	0.387
Peripapillary	57.94 ± 3.6	58.50 ± 3.0	57.31 ± 4.1	0.445
Superior-hemi	58.49 ± 3.8	59.11 ± 3.2	57.78 ± 4.3	0.259
Inferior-hemi	57.34 ± 3.7	57.82 ± 3.1	56.80 ± 4.2	0.728
**RPC capillary density (%), mean** **±** **SD**				
Mean	48.83 ± 2.9	49.33 ± 2.5	48.26 ± 3.2	0.257
Peripapillary	51.24 ± 3.6	51.79 ± 3.2	50.62 ± 4.0	0.272
Superior-hemi	51.51 ± 3.9	52.11 ± 3.4	50.82 ± 4.3	0.101
Inferior-hemi	50.94 ± 3.7	51.43 ± 3.2	50.38 ± 4.1	0.640
**Macular VD (%), mean** **±** **SD**				
Mean SCP	48.88 ± 3.3	49.85 ± 2.4	47.77 ± 3.7	0.024^*^
Parafovea SCP	51.17 ± 3.8	52.56 ± 2.7	49.58 ± 4.3	0.026^*^
Superior hemi in parafovea SCP	51.37 ± 3.8	52.88 ± 2.7	49.64 ± 4.2	0.036^*^
Inferior hemi in parafovea SCP	50.97 ± 4.1	52.25 ± 3.0	49.51 ± 4.6	0.026^*^
Mean DCP	49.31 ± 5.5	50.10 ± 5.2	48.41 ± 5.7	0.677
Parafovea DCP	53.77 ± 4.1	54.37 ± 4.0	53.09 ± 4.2	0.467
Superior hemi in parafovea DCP	54.15 ± 4.2	54.79 ± 3.9	53.42 ± 4.3	0.369
Inferior hemi in parafovea DCP	53.40 ± 4.3	53.95 ± 4.2	52.77 ± 4.3	0.608
FD300	52.96 ± 4.4	54.07 ± 3.9	51.70 ± 4.6	0.283

*CTO, coronary total occlusion; OR, odd ratio; CI, confidence interval; RNFL, retinal nerve fiber layer; RPC, radial peripapillary capillary; VD, vessel density; FD, foveal density; LVEF, left ventricular ejection fraction; hs-TnT, high-sensitivity troponin T; NT-proBNP, N terminal pro-brain natriuretic peptide*.

* *p < 0.05 is considered statistically significant*.

† *Adjusted for sex, age, total cholesterol (TC), high-density lipoprotein cholesterol (HDL-C), history of BP grade, history of diabetic mellitus, smoke, LVEF, hs-TnT, NT-proBNP, and Gensini score*.

### Multivariate Regression Analysis and ROC Analysis

We sought to study the potential value of retinal vasculature parameters in discriminating CTO from non-CTO group. Multivariate regression analysis in Table 3 exhibits a positive relationship between the presence of CTO with clinical variates: sex (male, OR, 5.609; 95% CI, 1.181–26.641; *p* = 0.003), LVEF (OR, 0.916; 95% CI, 0.880–0.954; *p* < 0.001). Besides, multivariate regression analysis also exhibited a positive relationship between the presence of CTO with VD of superior hemi in the parafovea SCP (OR, 0.772; 95% CI, 0.685–0.870; *p* < 0.001).

**Table 3:** Risk factors for CTO using logistic regression analysis.

**Variates**	**Univariate model**		**Multivariate model**	
	**OR (95% CI)**	***p-*value**	**OR (95% CI)**	***p-*value**
**Sex**
Female	Reference		Reference	
Male	4.540 (1.651–12.486)	0.003^*^	5.609 (1.181–26.641)	0.030^*^
LVEF, %	0.916 (0.883–0.951)	<0.001^*^	0.916 (0.880–0.954)	<0.001^*^
NT-proBNP, pg/mL	1.001 (1.001–1.002)	<0.001^*^		
**Retinal VD of SCP, %**				
Mean	1.254 (1.135–1.385)	<0.001^*^		
Parafovea	1.300 (1.177–1.435)	<0.001^*^		
Superior hemi in parafovea	1.331 (1.202–1.474)	<0.001^*^	0.772 (0.685–0.870)	<0.001^*^
Inferior hemi in parafovea	1.209 (1.114–1.311)	<0.001^*^		

*CTO, coronary total occlusion; OR, odd ratio; CI, confidence interval; LVEF, left ventricular ejection fraction; NT-proBNP, N terminal pro-brain natriuretic peptide; VD, vessel density; SCP, superficial capillary plexus*.

* *p < 0.05 is considered statistically significant*.

**Figure 1:**
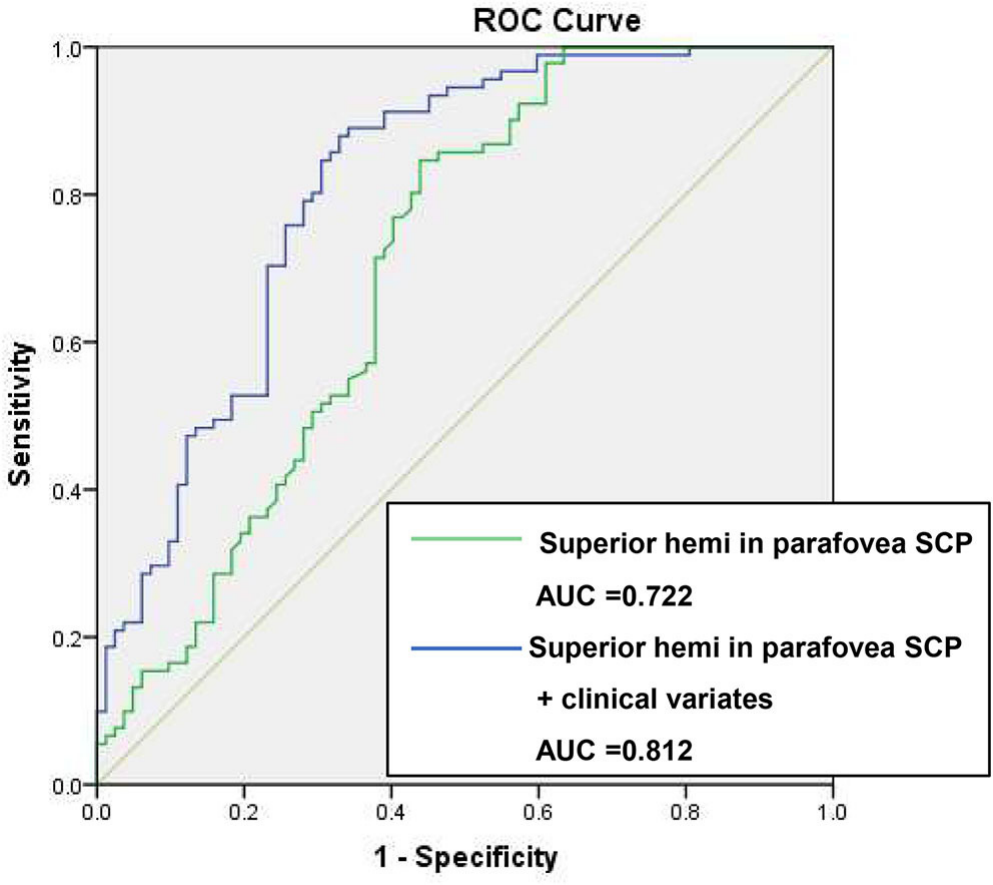
ROC curves of retinal vasculature and clinical variates in differentiating CTO from CAD. ROC, receiver operating characteristic; VD, vessel density; CTO, chronic total occlusion; CAD, coronary artery disease; AUC, area under the curve. Clinical variates refer to sex, left ventricular ejection fraction (LVEF).

**Figure 2:**
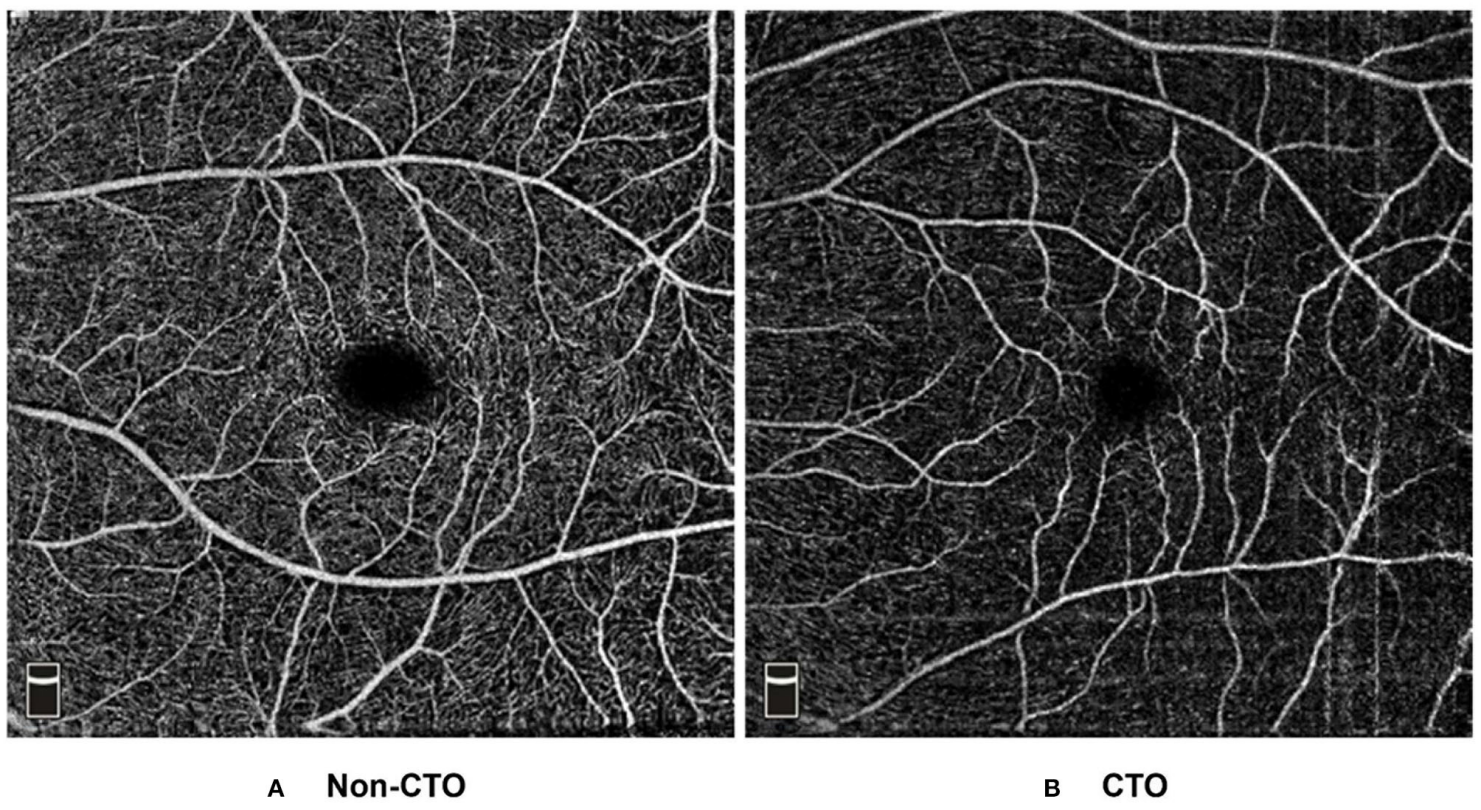
Example OCT angiograms of DCP in the non-CTO and CTO eyes. Typical 6 × 6 mm high-definition OCT angiograms in the SCP: **(A)** a non-CTO CAD eye with higher retinal VD (VD of mean, parafovea, superior hemi parafovea and inferior hemi parafovea in the SCP: 55.63, 61.45, 62.13, and 60.77%, respectively). **(B)** a CTO eye with lower retinal VD (VD of mean, parafovea, superior hemi parafovea and inferior hemi parafovea in the SCP: 36.55, 36.50, 38.37, and 34.62%, respectively). CTO, coronary total occlusion; SCP, superficial capillary plexus; VD, vessel density.

Based on the results of multivariate regression analysis, ROC curves were generated to show the prediction of CTO using the variates. The ROC curve of VD of superior hemi in the parafovea SCP showed the ability to predict CTO from CAD with an AUC of 0.722 (specificity of 53.9% and sensitivity of 86.2%). When VD of superior hemi parafovea SCP was combined with the selected clinical variates (sex and LVEF), the AUC increased to 0.812 (specificity of 89.0% and sensitivity of 65.9%) (see Figure 1). Figure 2 shows the OCTA images of SCP in non-CTO and CTO groups.

## Discussion

Our study demonstrated the significant difference of retinal vasculature differences in CTO patients when compared to non-CTO CAD patients. Our findings highlighted two important discoveries: one was the link of the retinal microvascular changes and CTO, and the other was that a surrogate of retinal vasculature can provide incremental value as damage of microvasculature and assist in the assessment of CTO.

CAD patients were found to present with narrowing retinal arterioles (8, 10) and decreased VD in the SCP and DCP (11). An early hypothesis had declared that retinal arterioles could reflect the microvascular circulation status (19). The narrowing retinal arterioles might result from microvascular damage, such as the process of aging, hypertension, and from the intimal thickening, medial hyperplasia, hyalinization and sclerosis (20, 21). Another hypothesis might be related to the endothelial dysfunction coexisted in CAD patients. Importantly, the process of atherosclerosis begins early in life, and endothelial dysfunction contributes to atherogenesis (22). Endothelial dysfunction is a systemic condition and has been detected in the coronary epicardial as well as in peripheral arteries (23), in which peripheral endothelial function was found to be associated with coronary artery endothelial function (24). Since subtle changes in the retinal vasculature might mirror preclinical information useful for predicting clinical cardiovascular events (20, 25), retinal microvasculature could thus offer a readily accessible window to assess the microcirculation.

In this study, SCP were found to be significantly decreased in the CTO patients when compared to non-CTO CAD patients. SCP is a network with both large and small vessels directly connected to the retinal arteries and veins, and supplies all the other vascular plexuses (17). The end-artery system of the retina lacks autonomic nerve supply, and blood flow into the capillary beds is tightly regulated in response to the metabolic needs of the retinal parenchyma (26). This autoregulation is achieved mostly by smooth muscle cells (SMCs) in the retinal arteries and arterioles, which are located in the SCP. SMCs are highly sensitive to endothelial-generated vasodilators and vasoconstrictors, helping maintain a constant retinal blood supply (27). Impairment of autoregulatory function in CAD patients has been stated associated with the severity of coronary arteries occlusion (28). Therefore, CTO patients might suffer from much severer impairment of autoregulatory function in retinal vessel SMCs, which leads to lower VD in the SCP.

Decreased cardiac output could be another possible reason contributed to the lower VD of SCP in CTO patients. In multivariate regression, LVEF was shown to be a positive risk factor in predicting CTO. A previous study has also shown decreased VD in the SCP in acute coronary syndrome patients presented with lower LVEF (12). LVEF is one of the most important parameters in the assessment of left ventricular systolic dysfunction (29) and it may be associated with retinal arterial caliber in left ventricular remodeling (30). Moreover, perfusion of the ophthalmic artery may be decreased when cardiac output is compromised (31). Further studies are still needed to confirm the association between the impaired retinal VD and hemodynamic parameters to reflect cardiac output.

There was no significant difference of VD in the DCP between CTO and non-CTO patients. DCP contains capillaries of uniform size, without larger vessels that interconnect the plexuses (17). Each capillary unit consists of a continuous endothelium surrounded by pericytes (26). Retinal endothelial cells and pericytes possess autoregulation properties and help maintain a stable blood flow through the capillaries in hypoperfusion of the retinal arterioles (32). A possible explanation might be that the association between retinal circulation and incident CTO might be a vascular process that more specifically targets to arterioles than capillaries.

Our study generated several ROC curves to evaluate the performance of retinal vasculature in discriminating CTO from non-CTO CAD. The ROC curve showed a satisfying result of AUC with both considerable sensitivity and specificity when combining the most predictive retinal vasculature parameter and clinical variates. The result indicated the parameter of superior hemi in the parafovea SCP possessed a good characteristic to differentiate CTO from non-CTO CAD patients. As previously documented (33), arteriolar narrowing was correlated with gravity inversion. Besides, arteriolar diameter changes were thought to be a compensatory response to microgravity, where in intravascular and extravascular body fluids shift under the absence of the hydrostatic gradient (34). Therefore, we speculated that vessels on the superior macular area might be much more sensitive to this mechanism and present with narrower arteriolar caliber (35) and lower retinal VD.

The finding of a significant link of the decreased retinal VD in the CTO patients had potential implications, particularly for those CAD patients with risks to develop CTO. In fact, CTO is a condition frequently encountered in the catheterization operating theater with a prevalence up to 20% among patients clinically indicated for CAG (36). The retina provides a non-invasive window to detect the development and progression of coronary arteries disease. Our results suggested that the changes of retinal VD were typically characterized in the SCP rather than DCP in CTO patients, the surrogate of the VD in the SCP could be an indicative of microvascular damage for CTO. Since the diagnosis of CTO is still made based on CAG, our study might offer a convenient and non-invasive method for CAD patients to assess the process of coronary artery lesions based on OCTA. In such cases, OCTA might work as a screening tool in the detection of CTO, and it could improve the patient selection before compromising a diagnosis.

Strengths of this study included the non-invasive, quantified retinal vasculature measurement and direct CAG assessment. We acknowledged several limitations in our research. This was a cross-sectional study, and a follow-up survey might be more helpful in assessing the retinal vascular changes. Besides, there might be not-everywhere availability of OCTA. In addition, the number of patients seemed small, and a large sample would be preferable.

It remains to be confirmed whether OCTA or retinal vasculature could add incremental predictive value beyond the traditional cardiac characteristics in a practical manner. Our study is a pilot study to evaluate the retinal microvasculature in CTO patients. Further study is still ongoing to explore the clinical practicability of retinal vasculature in helping with assessing the long-term cardiovascular outcomes in CTO patients.

## Conclusions

Our study results indicated that retinal VD was significantly decreased in CTO patients, and the microvascular damage might specifically target to arterioles than capillaries in CTO. Retinal vasculature could thus be a surrogate for detecting the microvascular damage and assist in the assessment of CTO. The independent associations observed between the retinal VD and the CTO supported the view that retinal vasculature signs might reflect the lifetime cumulative effects of the vascular process on the microvasculature (19).

## Data Availability Statement

The raw data supporting the conclusions of this article will be made available by the authors, without undue reservation.

## Ethics Statement

The studies involving human participants were reviewed and approved by Research Ethics Committee of Guangdong Provincial People's Hospital. The patients/participants provided their written informed consent to participate in this study.

## Author Contributions

PZ, HY, and XY: study concept and design. PZ and YH: drafting of the manuscript. LJ, NT, HY, and XY: critical revision of the manuscript for important intellectual content. PZ, QP, MH, CL, and YK: statistical analysis. HY and XY: obtained funding. PZ, YH, LJ, NT, HY, and XY: administrative, technical, and material support. NT, HY, and XY: study supervision: All authors: acquisition, analysis, interpretation, contributed to the article and approved the submitted version.

## Funding

This work was supported by Project of Special Research on Cardiovascular Diseases (2020XXG007; XY); Science and Technology Program of Guangzhou, China (202002020049; XY); Project of Investigation on Health Status of Employees in Financial Industry in Guangzhou (Z012014075; XY); the National Natural Science Foundation of China (81870663; HY); the Talent Introduction Fund of Guangdong Provincial People's Hospital (KJ012019087 and Y012018145; HY).

## Conflict of Interest

The authors declare that the research was conducted in the absence of any commercial or financial relationships that could be construed as a potential conflict of interest.

## Publisher's Note

All claims expressed in this article are solely those of the authors and do not necessarily represent those of their affiliated organizations, or those of the publisher, the editors and the reviewers. Any product that may be evaluated in this article, or claim that may be made by its manufacturer, is not guaranteed or endorsed by the publisher.
